# Water Transport Dynamics and Kinetic Equilibria in Nanoblisters at the Graphene–Mica Interface

**DOI:** 10.1021/acs.langmuir.4c03622

**Published:** 2025-02-04

**Authors:** Joshua S. Roys, Nicholas D. Stucchi, Jennifer M. O’Brien, Adam D. Hill, Ryan D. Brown

**Affiliations:** † Department of Chemistry & Biomolecular Science, Clarkson University, Potsdam, New York 13699, United States; ‡ Department of Chemistry, Trinity College, Hartford, Connecticut 06106, United States

## Abstract

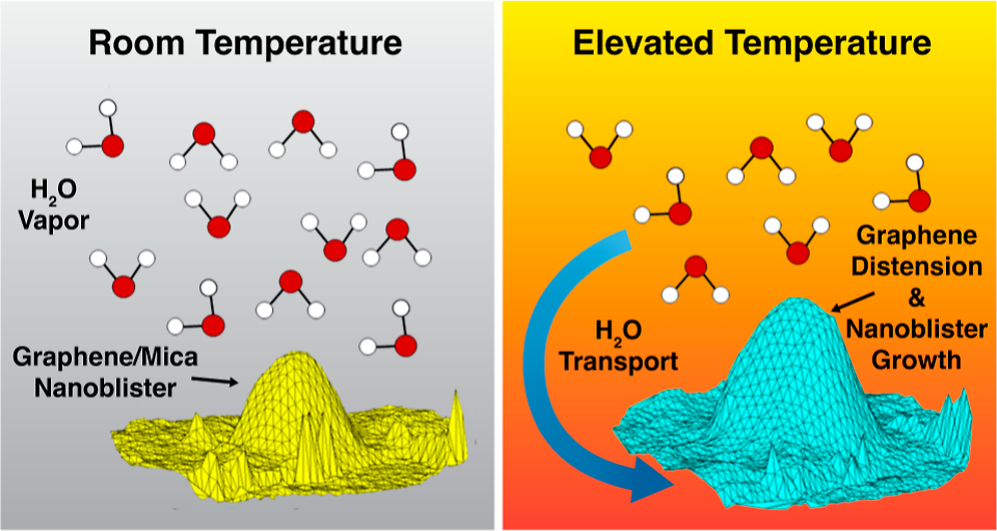

Nanoscale reduced volumes with novel properties can be produced from 2D materials like graphene. Mild thermal annealing imposes vast and varied amounts of water intercalation into the graphene–mica interface, resulting in the formation of nanoblisters and impacting the local environment for applications such as reactions confined at the solid–solid interface. Atomic force microscopy imaging (AFM) and micro-Fourier transform infrared (micro-FTIR) spectroscopy characterization after 60–120 °C anneals revealed large volumes of water readily intercalate into graphene–mica nanoblisters, elucidating water transport behavior under mild reaction conditions. The inflation and deflation of graphene nanoblisters throughout the annealing process is attributed to the contraction of the graphene capping layer upon cooling from the annealing temperature, due to the independence of nanoblister aspect ratios from nanoblister volume or surface area. The intercalated water volume was estimated by the distended volumes of each nanoblister and exhibit an equilibrium trend established after 2 h of annealing. This water equilibrium occurs at a variety of temperatures, but higher temperatures favor graphene contraction and distention to accommodate larger volumes of water. Nanoblister volumes are set during the cooling process, indicating a kinetic trapping effect that can influence physical properties and reactivity for all systems confined at the graphene–mica interface.

## Introduction

**Figure 1:**
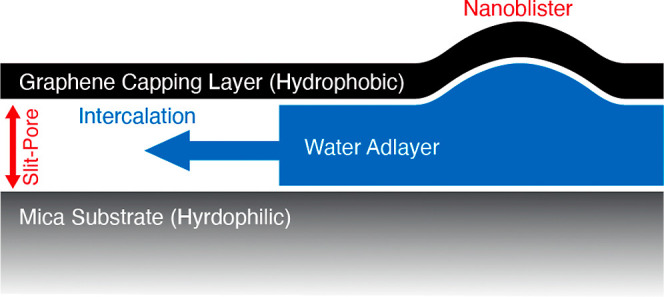
Water intercalated between graphene and mica can occupy either the slit-pore or large nanoblister volumes.

Restricted nanoscale volumes show unique regimes of reactivity and phase behavior. When nanoscale volumes are produced under ambient conditions, interfacial water contributes to the physical and chemical properties of materials with impacts on reaction pathways, surface wettability, and solvation and functionality of proteins and other biological molecules.^1−5^ Capping surfaces with exfoliated 2D materials like graphene (a “solid–solid” interface) has become a facile and widely employed technique to produce these reduced volumes, making it particularly critical to understand the behavior of water in these settings.^6,7^ Water confined beneath graphene can sequester into one of two volumes: the 2D nanochannel “slit-pore” or the nanoblisters that extend above the surface (Figure 1). While the former is a well-established standard in the characterization of confined water and has been extensively characterized by techniques like atomic force microscopy (AFM), transport in nanoblisters have been less well-explored and sometimes treated as aberrant.^8−10^ Nonetheless, recent work has shown that these volumes can contribute meaningfully to reaction outcomes or serve as nanoreactors.^11−13^ Using AFM and micro-Fourier-transform infrared (micro-FTIR) spectroscopy, we have characterized the volume, morphology, and composition of nanoblisters at the graphene–mica interface following an array of annealing conditions and demonstrated that nanoblisters can kinetically trap significant and highly variable amounts of water that reflect the annealing conditions.

Kinetic trapping occurs when a system reaches a metastable state separated from thermodynamic equilibrium by an energetic barrier.^14^ When the system is heated or otherwise excited to an appropriate energy, molecules pass over the barrier; when the temperature is lowered, molecules no longer possess the energy to frequently cross the barrier and remain trapped, even when it is more thermodynamically favorable for the molecules to exit the metastable state. The result is a system with memory, exhibiting a distribution of molecules reflective of the previous higher-temperature/energy conditions experienced.^15^ This behavior is observed not only in surface adlayers, but also supports the structures of proteins and supramolecular assemblies.^16−21^ Understanding the conditions under which a system enters a kinetically trapped state informs the subsequent stability and reactivity of the system; assuming that only a thermal equilibrium exists will result in the neglect of key dynamics.

The hydrophobic–hydrophilic graphene–mica interface is a well-studied system, which most prior work focusing on the slit-pore and the reversible wetting and dewetting behaviors in response to changes in relative humidity (RH).^6−8,22−27^ Wetting dynamics were previously studied as the slit-pore restored equilibrium following perturbations to the relative humidity, observing the dendritic growth of initial intercalated water domains.^8,23,26,28,29^ This sensitivity to ambient conditions suggests that, in concert with temperature changes, significant water volumes might preferentially occupy and subsequently become trapped in nanoblister features. Previous studies showed the effects of temperature on confined partial water layers that remained trapped at the graphene–mica interface even when exposed to UHV and temperatures of 600 °C.^24,25^ The existence of this apparently permanently trapped water suggests that water confined at the graphene–mica interface represents a kinetic equilibrium, as those conditions would otherwise result in total dehydration of the interface. However, the body of published work lacks an experimental determination of the relationship between temperature and water transport as they connect to the behavior of graphene–mica nanoblisters.^9,30−33^

Recent work has shown that water in nanoblisters can contribute meaningfully to ambient conditions for reactions taking place within the graphene–mica interface, including the growth of covalent organic frameworks via dehydration reactions in which nanoblisters serve as a reservoir of water to facilitate enhanced crystallinity.^11^ This work, in concert with other works describing enhanced or anomalous reactivity within graphene nanoblisters or nanobubbles, suggests that an expanded understanding of nanoblister behaviors is key to predicting and explaining reactivity at the solid–solid interface.^12,13,34,35^ We investigated water transport in and out of graphene–mica nanoblisters using AFM to characterize blister volume and morphology and micro-FTIR to characterize blister contents. Large-scale water diffusion was observed with volumetric trends suggesting the amount of confined water varies with annealing time and occurs at annealing temperatures as low as 60 °C. Further analysis of the total flake areas, nanoblister surface areas, and aspect ratios also indicate the thermal annealing causes contraction of the graphene flakes and changes in nanoblister morphology. These results inform the development of graphene-capped systems at ambient conditions and serve as a model of water transport in a variety of other reduced-volume systems.

## Experimental Section

### Materials and Sample Preparation

Mica substrates used were SPI Supplies V-5 grade muscovite mica, and graphene flakes were exfoliated from SPI Supplies highly ordered pyrolytic graphite (HOPG) substrates of SPI-2 grade. All mica substrates were tape-cleaved prior to sample preparation. The mechanical exfoliation of graphene was accomplished by briefly exposing the mica substrate to firm contact with the HOPG twice in succession. Exfoliation was performed at ambient humidity of 20–60% for initial 120 °C isothermal and temperature series samples, and following the evaporation of a 10 μL milli-Q water dose for all other samples to compensate for low ambient RH (≈10–15%). The resulting few-layer graphene flakes were located for AFM imaging using the optical contrast observed between the flakes and mica substrate under a 10× objective optical microscope.

**Figure 2:**
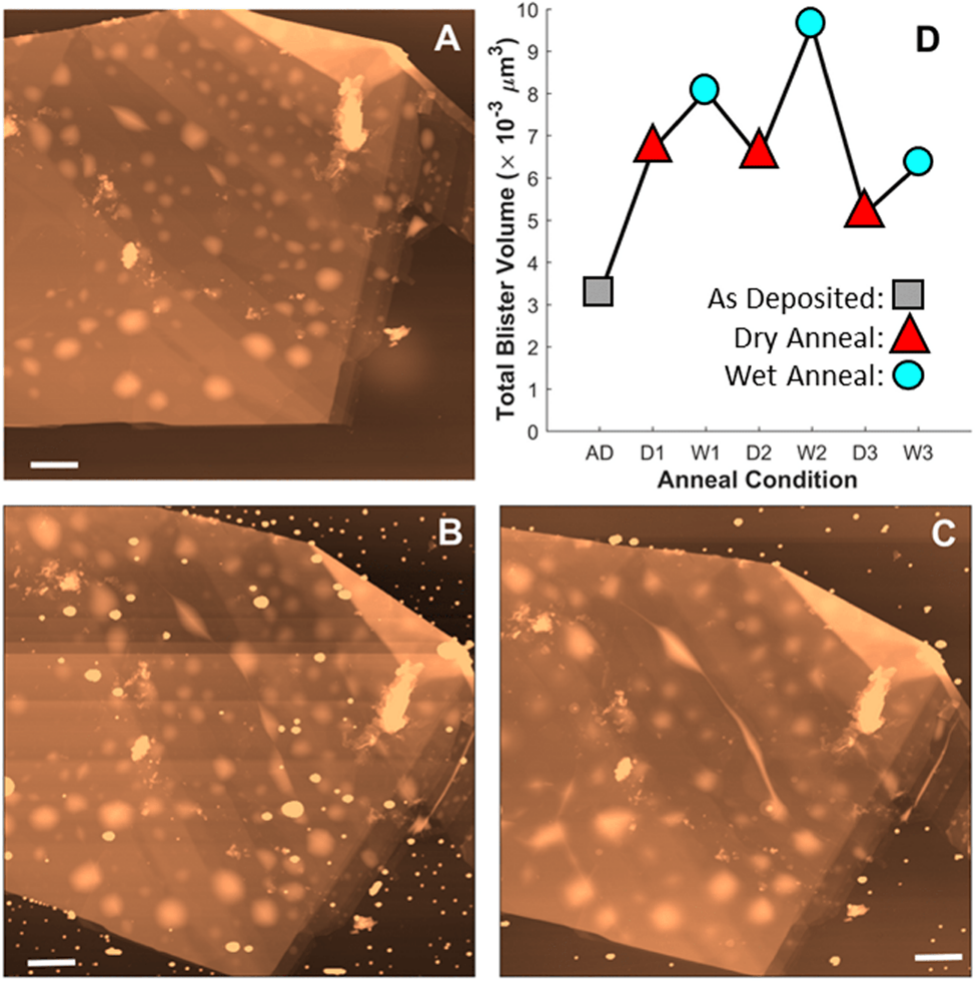
AFM topography images (10 μm × 10 μm) of a flake following a series of dry and wet annealing steps (A–C). Flake morphology is presented as deposited (A), after a “dry” 120 °C anneal (B), and after a “wet” 120 °C anneal (C). All scale bars are 1 μm. Plot (D) illustrates the total volume of water confined in nanoblisters for each step after cycling twice more between dry and wet anneals.

**Figure 3:**
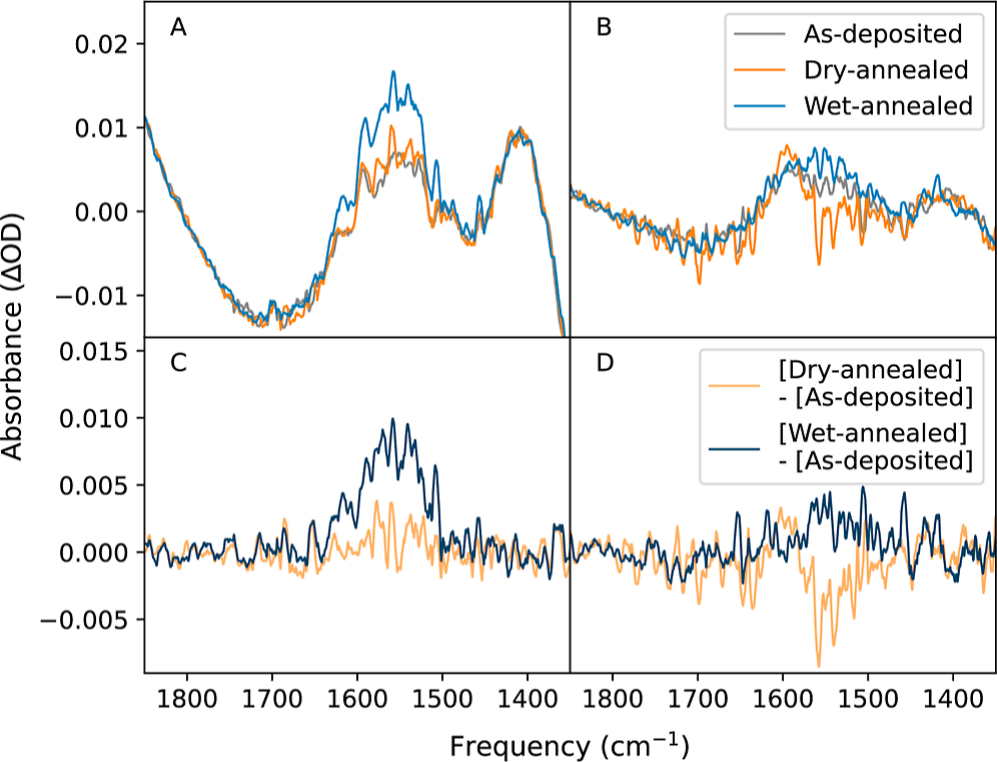
(A,B) FTIR difference spectra of exposed areas subtracted from spectra taken on flakes 1 and 2 in Figure S6, respectively. (C,D) FTIR difference spectra for flakes 1 and 2 in Figure S6, respectively, with the as-deposited spectra subtracted.

**Figure 4:**
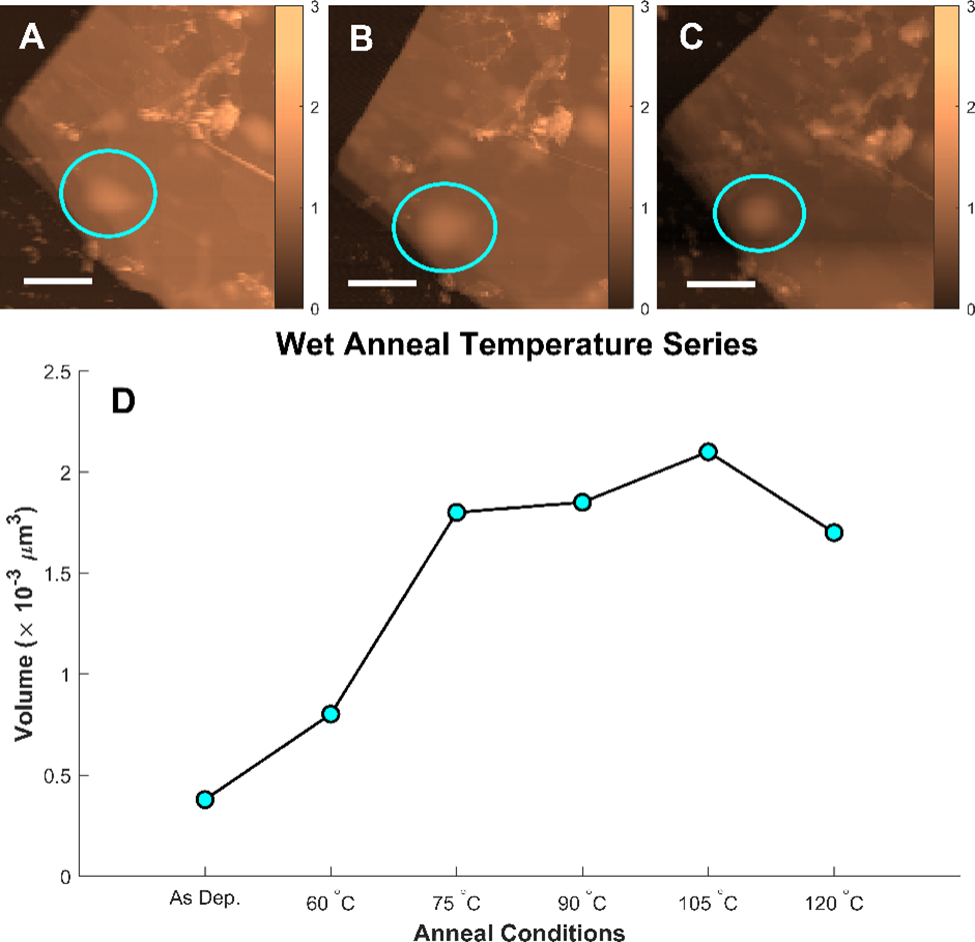
(A–C) AFM topography images (4 μm × 4 μm) of a few-layer graphene flake at room temperature, 75, and 120 °C, respectively. All scale bars are 1 μm, and blue circles indicate examples of nanoblisters. Color bars are in units of nm. (D) Total volume of water confined within all nanoblisters across the flake for each step of a varied temperature annealing series.

### Sample Annealing Details

Closed system (“wet” anneal) samples were annealed in a 15 mL glass pressure vessel (Chemglass, #15 thread) sealed by a polytetrafluoroethylene (PTFE) cap and O-ring. The pressure vessel was heated in a Thermo Scientific benchtop muffle furnace (model no. F48050). Water reservoirs sufficient to achieve 100% RH were added to the bottom of wet anneal sample vessels as either 0.1 g CuSO_4_·H_2_O or 75 μL milli-Q water.^36^ Open system (“dry” anneal) samples were annealed in the glass vessel without the PTFE cap and heated in a vacuum oven (Fischer Isotemp model 280A) under house vacuum at approximately 200 Torr.

### Characterization Methods

All AFM experiments were performed at room temperature under ambient atmosphere using a Bruker MulitMode VIII AFM. Antimony (n) doped silicon cantilever tips were used (Value AFM Probes Model VNCHV-A or Ted Pella, Inc. TAP-300-ALG). All images were collected in tapping mode and processed in MATLAB using the Image Processing Toolbox and Statistics and Machine Learning Libraries as well as custom MATLAB scripts. A detailed description of the calculations and analysis, including the extraction of blister volumes from AFM images, can be found in the Supporting Information.

Micro-Fourier transform infrared spectroscopy was performed using a Thermo Nicolet 4700 FT-IR microscope equipped with a Triton 10× objective, WF10× −18 mm eyepiece, and MCT detector. The region from 4000 to 650 cm^–1^ was measured with 1 cm^–1^ resolution. Mica sheets were placed on a silver-coated aluminum slide and measured in reflectance geometry. A freshly cleaved mica sheet was used to blank the spectra. AFM-characterized flakes were repeatedly located before and after each anneal and compared with adjacent uncovered regions. Background spectra were collected at 512 scans and all others were collected at 128. The resulting data were analyzed using custom Python 3.9 scripts written in the Spyder v.5.3.3 IDE. Spectra were processed with a 25-point second-order (parabolic) Savitzky–Golay filter to remove perturbations from IR-active atmospheric gas signals. Further details regarding data processing are available in the Supporting Information.

## Results and Discussion

Water transport was investigated using samples of graphene mechanically exfoliated on mica at ambient conditions (room temperature at RH of 10–60%) which were imaged via AFM before and after annealing cycles. Exfoliated graphene closely conforms to surface features and tape-cleaved mica substrates provide large atomically flat terraces optimal for the exfoliation of graphene flakes away from mica step edges.^6,7,37,38^ Graphene flakes on the mica surface exhibit morphology consistent with trapped water under ambient conditions with greater than 30% RH.^9,22,23,25^ Most notably, flakes may form nanoblisters after exfoliation due to the low adhesion energy between water layers and the mica substrate.^39,40^ We observed water transport in and out of these water-filled nanoblisters by monitoring the changes in integrated volume of each blister after annealing under varied conditions. The volumes include only the distented graphene region of the blister, and do not include water adlayers present beneath each blister. We observed a mica–graphene step height of 0.3–0.8 nm, consistent with one monolayer of water present between the graphene and mica.^41^

Water transport was characterized by AFM images of the water-filled graphene nanoblisters, the volumetric analysis of these nanoblisters, and subsequent characterization by micro-FTIR spectroscopy. These measurements confirm the intercalation of H_2_O into the graphene–mica slit-pore, which occurs on the scale of ≈10^–4^ μ m^3^ H_2_O per μ m^2^ graphene following thermal anneals at and above 60 °C. To define the equilibrium relationship between confined and external water, the behavior of confined water with respect to temperature was investigated comparing blister geometry and size before and after sample annealing. Blister volumes exhibited a recurring plateau after 2 h of annealing, observed across the full range of annealing temperatures. As all AFM imaging was performed at room temperature, the stability of blister volumes across several heating and cooling periods indicates water trapped in a kinetic state defined by potential barriers greater than the thermodynamic driving force for water transport in or out of graphene confinement. Assuming the kinetic states are populated by water unable to escape the potential well during cooling, the trapped water volume is likely tunable through the cooling rate and therefore the annealing temperature.

Intercalated water showed a preference for nanoblisters over the slit-pore. When annealed in an open container under vacuum conditions at 120 °C, the total volume of nanoblisters more than doubled. Blister growth in the absence of external water vapor is attributed to the consolidation of water trapped during the exfoliation process, as evidenced by the disappearance of various morphological structures after the anneal (Figure S3). When an external water source is added to the closed reactor, the 120 °C anneal further increased the volumes of the same nanoblisters. Blisters had already consolidated prior to the anneal from water trapped by the initial flake exfoliation, and mica substrate dehydration occurred mainly along the basal plane as opposed to bulk-to-surface diffusion, so the additional water most likely entered through the graphene–mica slit-pore.^32,33^ Similar behavior has been observed at the graphene–SiO_2_ interface, reportedly due to the rough nature of the SiO_2_ substrate after long-term exposure to high RH.^30^ The graphene–mica interface in the same study exhibited water intercalation only at the graphene edge, and did not report changes in nanoblister formation or morphology. The nanoblister evolution observed under elevated temperatures implies that kinetic constraints, rather than thermodynamic constraints, govern water intercalation. The dynamic changes in nanoblister shape and size with each annealing step points to a kinetic process that dictates the volume and structure of water confined by the graphene flake, instead of a consistent morphology expected from a thermodynamic state.

Repeated cycling between dry and wet anneals at 120 °C resulted in Figure 2 below, with wet anneal volumes consistently higher than in dry anneals. The higher wet volumes are indicative of water transport in and out of the graphene flake edges, and the shift in volume between each annealing step constitutes a substantial amount of water on the order of 10^–3^μ m^3^.

To further ensure the volume change was due to the movement of water, micro-FTIR spectra were collected for samples as-deposited, after a 120 °C dry anneal, and after a 120 °C wet anneal. AFM optical micrographs (Figure S5) were referenced to collect FTIR spectra of the same AFM-imaged flakes for direct comparison of water content observed in both techniques. These flakes used for FTIR characterization exhibited standard blister behavior as well as the storage of intercalated water within graphene creases. The water-filled creases followed the same trend as the nanoblisters, likely obeying a similar diffusion mechanism.

A background micro-FTIR spectrum from the blank mica adjacent to each few-layer graphene flake was subtracted to create difference spectra between the graphene-confined and exposed regions shown in Figure 3a,b. The subtraction process removes signals from varying mica thicknesses, and interference patterns from mica layer interactions. The O–H bending mode, centered at 1560 cm^–1^, was most easily observed; strong mica absorbances made measurements in the O–H stretching region unreliable. In agreement with the AFM data, more water is observed beneath the flakes than outside, and wet annealing resulted in higher water content. Further subtraction of the initial as-deposited spectra from subsequent postanneal spectra also removed graphene signals to isolate the confined water signal (Figure 3c,d). The water peak intensity increased significantly after wet annealing, while dry anneals minimally changed in intensity. The greater water intensity observed in wet annealed samples demonstrates the higher influx of water into the flake in the presence of a hydrated external environment as opposed to a dry environment under vacuum conditions. Dry annealed samples did however exhibit a blueshifted water peak around 1598 cm^–1^, prominent in Figure 3d, which is likely due to an increased presence of bulk-like water instead of interfacial water in contact with the graphene capping layer.^42−45^ An increase in bulk-like water suggests a surface-area-to-volume ratio change from dry annealing, in agreement with the consolidation of confined water into nanoblisters during dry anneals (Figure S3).

The influence of various temperatures on water intercalation was observed by annealing a sample from 60 to 120 °C in 1 h increments at 15 °C, while maintaining the same relative humidity within the reactor. The RH was held constant at 100% by addition of 75 μL of milli-Q water to the reactor vessel prior to annealing. Results of the annealing series are shown in Figure 4 below.

**Figure 5:**
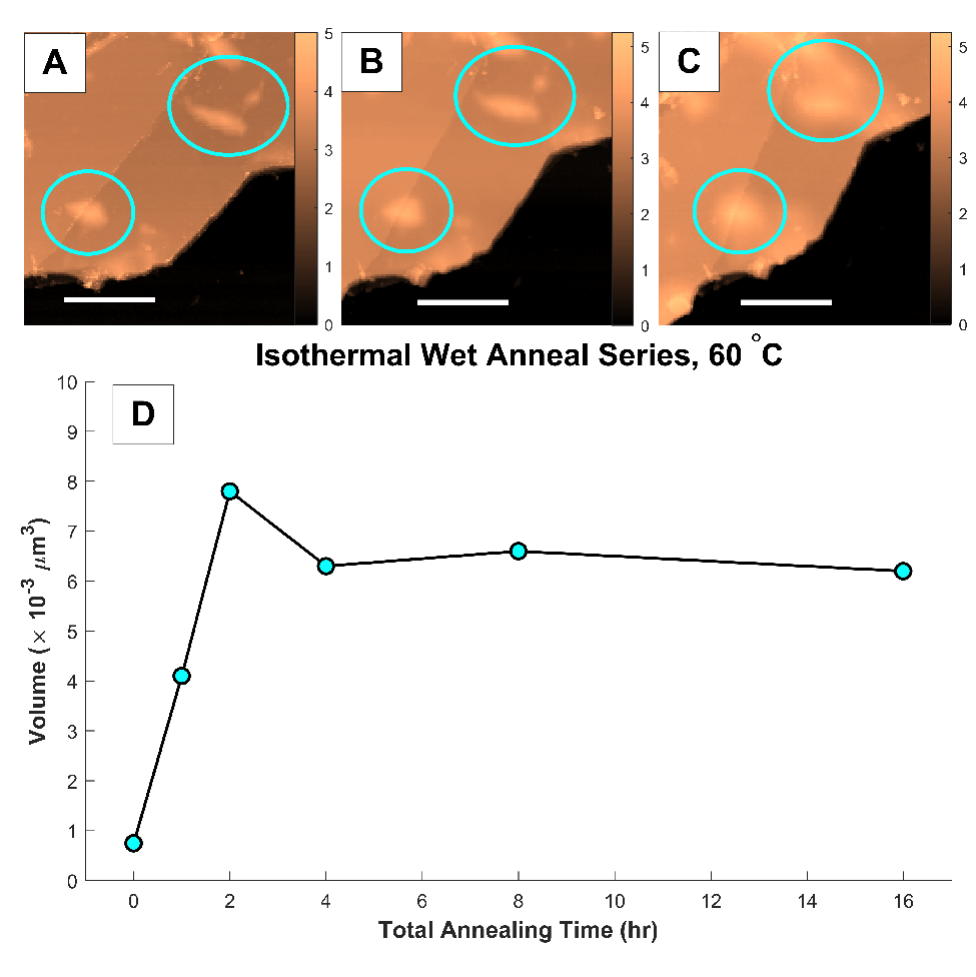
(A-C) AFM topography images (3 μm × 3 μm) of a few-layer graphene flake at room temperature, 60 °C after 2 h, and 60 °C after 16 h, respectively. All scale bars are 1 μm, and blue circles indicate examples of nanoblisters. Color bars are in units of nm. (D) Total volume of water confined within all nanoblisters across the flake for each step of an isothermal annealing series.

The blister nanovolumes depict a similar trend to previous wet anneals, resulting in confined volumes more than four times the as-deposited value due to the hydrated annealing conditions. Interestingly, the nanoblister volumes exhibit a plateau beginning at 75 °C after 2 h total annealing time. This establishes a nonlinear relationship between water intercalation and temperature, and is reminiscent of the plateau observed in Figure 2 which began after 2 h of annealing. To determine the effect of annealing time on water diffusion, we performed an isothermal series at 60 °C with individual anneal lengths varying from 1 to 8 h as seen in Figure 5. The partial pressure of water in the reactor was kept at 0.218 bar (100% RH) throughout the 60 °C annealing steps.

Despite the low annealing temperature, nanoblister volumes experienced a greater influx of water than previously observed in hotter anneals. After 2 h of isothermal annealing, nanoblister volumes were an order of magnitude larger than the as-deposited sample. Additionally, the plateau trend is reproduced following the 2 h annealing mark with volumes remaining relatively constant even after 16 total hours of annealing. This behavior implies an induction time occurs within the initial 2 h of exposure to a heated and hydrated environment, during which the interface is filled with water to a saturation point. Therefore, we conclude that above a certain threshold temperature the existence of a confined/external water equilibrium is independent of temperature, maintaining a kinetically determined constant volume after the first 2 h.

**Figure 6:**
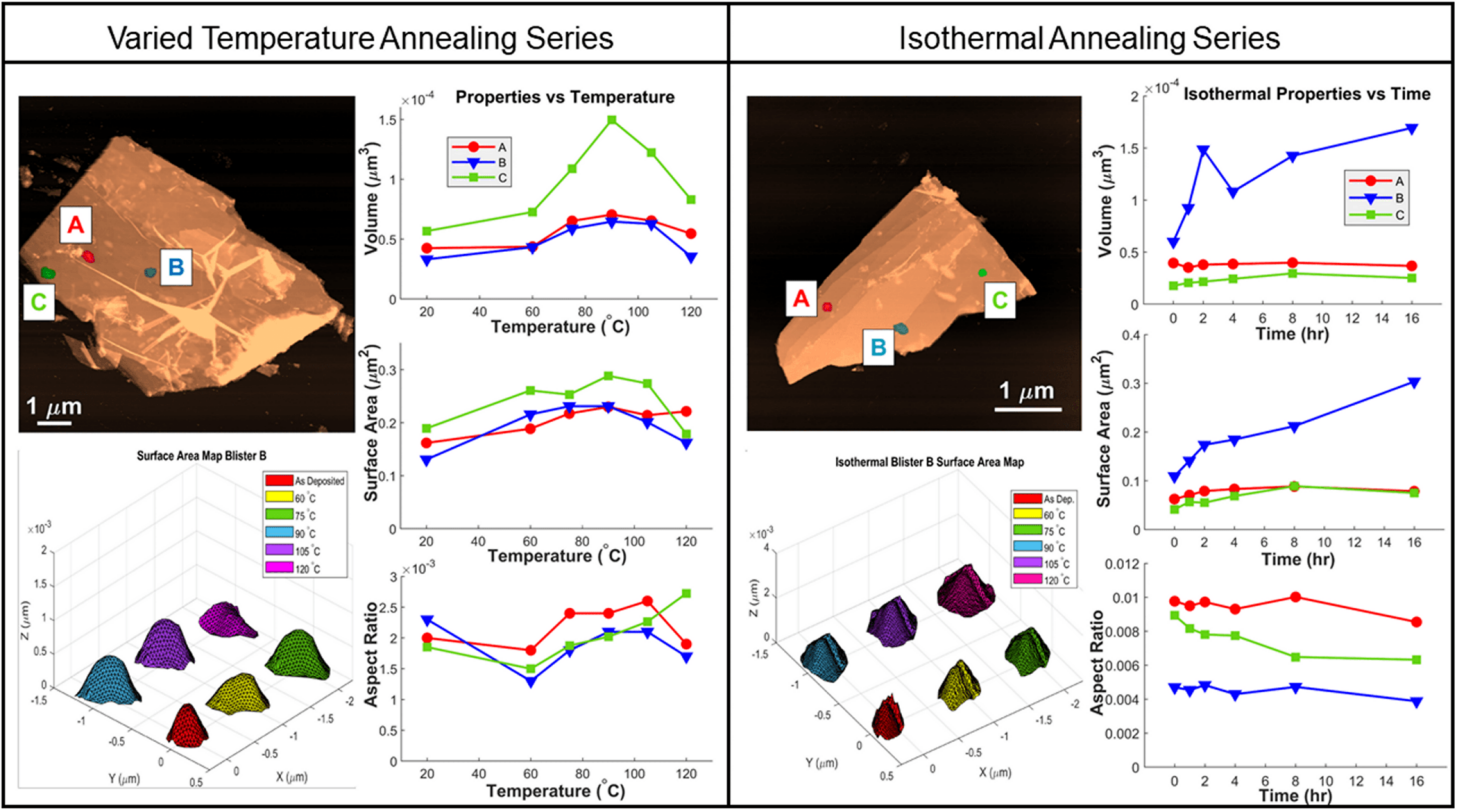
Individual analysis of nanoblisters in Figures 4 (left) and 5 (right) as indicated on the AFM topographic images. Results for the volume, surface area, and aspect ratio of blisters A (red), B (blue), and C (green) with respect to annealing temperature and time are displayed as line graphs. Surface areas were measured by constructing trimesh maps of each blister after each annealing step, as demonstrated for blister B of each flake in the bottom left panels.

Although the equilibrium exhibits temperature independence, observed changes in blister morphology imply elevated temperatures do impact the mobility of water confined by graphene. Blisters increased in size and fluctuated in shape throughout all previous annealing series, which may be attributed to increased water diffusion resulting from translational and rotational modes populated by the input of thermal energy in the system. These observations are consistent with the increased diffusivity reported in water/ethanol mixtures confined at the graphene–mica slit-pore when heated.^46^ Varying responsiveness of the graphene capping layer to water transport was also observed, affecting nanoblister growth and morphology. The changing appearances of these features throughout annealing processes may be due to differential pressures within the blisters, similar to the switchable shapes reported in graphene blisters on SiO_2_ substrates above and below a critical charging pressure.^47^ Examples of a water-filled nanoblister swelling and deflating are shown in Figure 6 where blister surface areas change dramatically throughout the annealing series. While the total nanoblister surface area of each flake after annealing was greater than the as-deposited surface area, individual blisters exhibit a wide range of surface areas at each annealing step which is not constrained to the overall trend in blister volume. The consistent changing of nanoblister surface areas and volumes between various annealing conditions further suggests that the system is dynamic during the annealing cycle, with considerable water transport occurring beneath the graphene flake during each anneal. This observation is also supported by the blueshifted peak shape change in the FTIR data.

Discrepancies in the behavior of individual blister surface areas and volumes compared to trends for the flake as a whole imply water transport beneath the graphene flake may be selective and favor some nanoblisters over others. This selectivity would account for the nonuniform growth and deflation of blisters among graphene flakes as well as the variety between FTIR spectra, and understanding the factors guiding water transport could facilitate a method for directing water diffusion at the graphene–mica interface. Studying the aspect ratios of relatively circular nanoblisters revealed that the blister aspect ratios vary nonlinearly with increasing temperature. Aspect ratios were calculated as *h*/*r* where *h* is the maximum blister height at the center of the nanoblister and *r* is the blister radius from that point to the nearest side.^39^ Aspect ratios for the blisters A, B, and C analyzed in Figure 6 varied by up to 77% between anneals. All of the blisters analyzed exhibited a decrease in aspect ratio when first annealed at 60 °C, followed by a gradual increase when annealed to higher temperatures as seen in Figure 6. Interestingly, the aspect ratios of the nanoblisters annealed isothermally at 60 °C in Figure 5 appear to be independent of both blister volume and blister surface area. The minimal changes in aspect ratio at constant temperature in Figure 6 suggest nanoblister morphology is a function of temperature, and water intercalation alone is insufficient to the distort the graphene capping layer to large degrees. The liquid-like confined water may simply adapt to the nanoblister volume following any change in the graphene distention upon cooling from annealing temperatures.

The erratic growth of nanoblisters throughout annealing processes raises the question of how graphene responds to the significant distention of the graphene layer by nanoblister evolution. One route to compensate for the distention is the stretching of C–C bonds to accommodate disturbances in the graphene layer morphology, as reported in the formation of corrugated graphene ripples in free-standing and substrate-mediated graphene.^48−51^ Another route is the contraction of the graphene flake, sliding along the substrate to increase the surface area of blisters at the cost of total substrate area confined by the flake. Previous work has shown that intercalated water can drastically reduce the friction experienced by graphene sliding on a mica surface, facilitating this latter route.^41,52^ Analysis of the flakes reported here reveals that following the initial anneal, the total coverage areas of most flakes were reduced by roughly 1–5% compared to their original area, as seen in Table 1 below. However, not all fluctuations in flake area are observed to be inversely proportional to the change in nanoblister volumes or surface areas at a given annealing condition which further suggests that water transport into swelling nanoblisters is not the direct cause of graphene contraction. Rather, it is possible water takes residence in distented graphene features already caused by thermal effects.

**Table 1:** Change in Total Nanoblister Volume, Total Nanoblister Surface Area, and the Projected Flake Area Between as Deposited Samples and Specified Annealing Conditions^a^

image figure	anneal condition	Δ blister volume (μ m^3^)	Δ blister surface area (μ m^2^)	Δ flake area (μ m^2^)
Figure 4 1 h each	60 °C	4.22 × 10^–4^ (211%)	0.68 (14%)	–1.61 (−2%)
	75 °C	1.72 × 10^–3^ (553%)	3.64 (77%)	–0.74 (−1%)
	90 °C	1.42 × 10^–3^ (474%)	2.9 (62%)	–0.43 (−1%)
	105 °C	1.62 × 10^–3^ (527%)	1.86 (40%)	–1.34 (−2%)
	120 °C	1.32 × 10^–3^ (447%)	2.11 (45%)	–3.4 (−5%)
Figure 5 isothermal, 60 °C	1 h total	3.35 × 10^–3^ (549%)	0.05 (4%)	–0.61 (3%)
	2 h total	7.05 × 10^–3^ (1043%)	0.54 (43%)	–0.43 (−2%)
	4 h total	5.55 × 10^–3^ (843%)	0.76 (61%)	0.38 (2%)
	8 h total	5.85 × 10^–3^ (883%)	0.84 (67%)	0.37 (2%)
	16 h total	5.45 × 10^–3^ (829%)	1.25 (100%)	0.04 (0.2%)
Figure S9 1 h each	60 °C	1.15 × 10^–2^ (302%)	23.6 (229%)	–1.78 (−1%)
	75 °C	2.44 × 10^–2^ (528%)	32.6 (317%)	–0.27 (−0.2%)
	90 °C	2.30 × 10^–2^ (504%)	35.1 (341%)	–4.62 (−4%)
	105 °C	2.42 × 10^–2^ (525%)	27.3 (266%)	–1.58 (−1%)
	120 °C	6.68 × 10^–2^ (354%)	6.6 (65%)	–3.15 (−2%)

a Percent changes relative to the as deposited flake are included in parentheses.

The observation of graphene flake areas “shrinking” is in agreement with work performed with graphene on SiO_2_ and Si_3_N_4_ substrates relating graphene flake contraction to compressive strain present upon cooling from a “sliding threshold” temperature (*T*_S_) to room temperature.^53^ Multiple reports attribute the origin of compressive strain in the graphene flake to the mismatch in coefficients of thermal expansion (CTE) between graphene and the substrate, causing the total mechanical strain in graphene to become negative when the system returns to room temperature.^53−55^ The sliding thresholds for monolayer graphene on SiO_2_ and Si_3_ N_4_ substrates were reported to be *T*_S_ = 390 K and *T*_S_ = 360 K, respectively. As SiO_2_ is a smoother substrate than Si_3_ N_4_,^56,57^ these results may indicate a correlation between greater surface roughness and a lower threshold temperature. However, this work was performed under vacuum conditions and does not account for intercalated water layers between the graphene flake and substrate. The effect of water layers and water-filled nanoblisters on the contraction of graphene at the graphene–mica interface may be significant, lowering the *T*_S_ to the annealing temperatures applied herein.

A lower sliding threshold is consistent with studies determining the CTE of single-layer (SLG) and multilayer graphene (MLG) floating on a water surface, where graphene flake contraction was observed within the range of 297–320 K.^58^ The CTE values calculated for MLG and SLG were found to be −0.4 and −5.8 ppm K^–1^, respectively, and with the weaker MLG contraction attributed to the reduction of transverse vibrations by interactions with adjacent graphene layers. Multilayer graphene’s resistance to contraction would explain the positive changes in flake area observed for Figure S9 in Table 1, which was comprised of more graphene layers than the other flakes analyzed. Therefore, we conclude that the large quantity of water between mica and the graphene capping layer—likely 3 or more layers thick within nanoblisters—may be sufficient to reduce the sliding threshold of single and few-layer graphene flakes below our lowest tested annealing temperature of 333 K. Volumetric changes in graphene nanoblisters may contribute to graphene sliding, but appear to be minimal.

## Conclusions

The volumetric analysis of water-filled nanoblisters at the graphene–mica interface indicates elevated-temperature conditions cause water to swell blisters by readily intercalating between the graphene capping layer and atomically flat mica. AFM imaging depicted the growth and inflation of nanoblisters following thermal annealing in hydrated environments, and micro-FTIR spectra of AFM-imaged flakes support the observed changes in volume and morphology with corresponding shifts in O–H bending mode intensities. All are consistent with increasing volumes of liquid water within nanoblisters following annealing. Based on trends in nanoblister volumes throughout isothermal, varied temperature, and dry/wet condition annealing series, we conclude that the equilibrium present between nanoblister-confined water and external water is kinetic in nature, trapping intercalated water in states defined by energetic barriers present during sample cooling. The kinetic equilibrium produces a saturated threshold volume after 2 h of annealing, which remained constant for up to 12 h of repeated heating and cooling between 60 °C and room temperature. Additionally, fluctuations in graphene flake area were observed and attributed to the sliding of graphene along the water-mica surface in response to compressive strain. The origin of the compressive strain is likely a mismatch in the coefficients of thermal expansion of graphene, confined multilayer water, and the mica substrate, although the intercalation of water into the graphene–mica interface may offer minimal contributions to the sliding behavior. Dramatic changes in nanoblister shape were also attributed to thermal effects, where blister aspect ratios were shown to significantly larger variation with changing temperatures compared to fluctuations in blister volume and surface area. Combined, these observations demonstrate that while large quantities of water diffuse into the graphene–mica interface at elevated temperatures, it is the annealing temperature which dictates the morphology of the graphene capping layer. This work advances the understanding of how water transport at the graphene–mica interface leads to the formation of large confined metastable water volumes, and informs further investigation of water behavior relevant to common experimental conditions above room temperature. These results can be applied to calculate the water content contributing to reactions performed at solid–solid interfaces, expand control over the saturation limit, and further the study of water transport between other atomically flat substrates and a 2D material capping layer.

## Supporting Information Available

Descriptions of AFM calibration, image processing, and computational analysis; micro-FTIR data processing details; additional figures showing AFM images through wet and dry anneals at various temperatures; optical micrographs showing positions of repeatedly measured flakes; surface area analyses of additional blisters (PDF)
