# Supplementary material for: Water Transport Dynamics and Kinetic Equilibria in Nanoblisters at the Graphene–Mica Interface
Source: Langmuir. 2025 Feb 4;41(6):3779–86. doi: 10.1021/acs.langmuir.4c03622 (PMC11841039; doi:10.1021/acs.langmuir.4c03622)
Supplement: Supplementary file 1 — la4c03622_si_001.pdf [file la4c03622_si_001.pdf]

Supporting Information: Water Transport Dynamics and Kinetic Equilibria in Nanoblisters at the Graphene–Mica Interface

Joshua S. Roys,† Nicholas D. Stucchi,† Jennifer M. O'Brien,† Adam D. Hill,*,‡ and Ryan D. Brown†

†*Department of Chemistry & Biomolecular Science, Clarkson University, Potsdam, NY 13699, United States*

‡*Department of Chemistry, Trinity College, Hartford, CT 06106, United States*

E-mail: adam.hill@trincoll.edu

Contents

Number of pages (including cover): 13

Number of figures: 12

Sample Preparation....S2

Atomic Force Microscopy....S2

Micro-FTIR Spectroscopy....S5

AFM Image Processing and Characterization....S6

References....S12

Sample Preparation

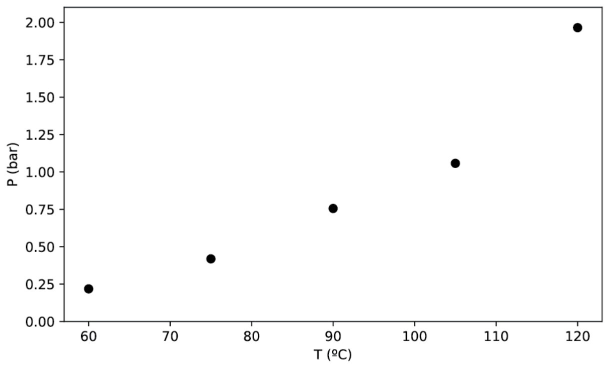

Figure S1: Saturation partial pressure of water as a function of temperature; wet anneals were performed at 100% RH, which corresponds to the partial pressure indicated at each temperature.[1]

Atomic Force Microscopy

AFM Calibration

Exfoliated graphene flakes were imaged at room temperature under ambient relative humidity (RH) which varied between 10 and 60%. An optimal microscope viewed the AFM sample stage to ensure the consistent analysis of individual flakes upon remounting the sample following any annealing process. Larger graphite flakes and debris features were used as landmarks to locate the analyzed flakes, also allowing relatively consistent rotational orientation of the sample. All images were internally calibrated against the known height of few layer graphene (FLG) steps present on FLG flakes, and strong contrasts in phase imaging confirmed the presence of graphene flakes on the mica surface. The graphene-mica step height varied between roughly 0.3 nm and 0.8 nm, well in agreement with the 0.4−0.9 nm range Heath et. al. attributed to the large chemical contrast between the graphene capping layer and the mica substrate.[2]

AFM Image Processing and Computational Analysis

All collected AFM data was interpreted both qualitatively and quantitatively using the MATLAB Image Processing Toolbox. Binary masks were the primary method for extracting quantitative information from the images, and were created using custom MATLAB scripts in a GUI allowing single-pixel precision. All AFM heights were analyzed by collecting the height at the mask pixel locations, allowing a comprehensive measurement of heights for the entirety of the selected features. Using this method, the average height of a masked feature provides a more reliable interpretation of feature height compared to traditional height profiles.

Internal calibrations for each image were performed *via* renormalization of the height data by a correction factor multiplied through each image pixel. The correction factor was generated for each image based on the apparent height of a FLG step within the image and was calculated as the quotient of the known FLG step height 0.335 nm divided by the observed step height (in nm). Only images height-corrected by the correction factor were used to collect quantitative data, and in the event that an image did not include an FLG step to calibrate to a correction factor from the same day collected under the same conditions was used. The AFM measurement uncertainty was determined to be roughly $\pm$ 0.03 nm by imaging atomically-flat fresh-cleaved mica, where the standard deviation of the image heights is representative of the measurement uncertainy present in all AFM images due to the instrumentation.

Following the height correction, binary masks of all the relevant image features were created by manipulating the image color scale to best visualize the desired feature and constructing the mask to adhere to the feature morphology. Once masked, quantitative data was extracted from the features by utilizing extensive custom MATLAB code to interpret the areas, volumes, and surface areas of the features. The script for each of these calculations is based on a flood-fill function applied to each selected feature on a Boolean representation of the input mask, expanding on the MATLAB 'imfill' function to individually bin the data

within any isolated mask feature. The individual data for each isolated feature- such as the water-filled nanoblisters analyzed here- allowed for the calculation of the feature area by the summation of pixels within the feature and conversion between pixels and micrometers. This method was also used to determine the total coverage area of graphene flakes imaged by masking the as a whole instead of specific features.

Nanoblister volumes were calculated individually for all selected isolated features and were collected in the form of an integrated volume. Every pixel within an image is square in shape and has a constant area 'a' defined by the scan size and pixel×pixel resolution of the image. Additionally, each pixel within a topograph possesses a height value 'h' corresponding to the image topography. Therefore the combination of each pixel and its height value can be considered a rectangular prism with the volume: $V = h \cdot a$. The volumes reported here are estimates resulting from the Riemann sum of all the rectangular prisms within a masked feature. All units for the lateral and height data are converted to μm prior to the calculation, producing volumes reported in μm^3. Additionally, the heights of each nanoblister feature are 'rezeroed' to begin at the minimum value of the the blister so as to interrogate only the volume confined by the distended graphene. The thickness of single layer graphene (0.335 nm) is also subtracted from each height to measure only the volume of the confined pocket, which is assumed to be fully filled with water. Subtracting the exact thickness of the graphene capping layer for few layer graphene was considered, but was determined negligible due to the minimal impact on volume observed by subtracting the single graphene layer.

The calculation of nanoblister surface area also utilized the custom flood-fill functionality to address isolated masked features and the height values of each pixel location. However for the surface area script all lateral information is converted to X and Y coordinates of μm units, and all height data (originally in nm) is converted to Z coordinates with μm units. Therefore (X, Y, Z) represents the coordinates of each point defining the surface topography of the masked feature, and these coordinates are used as the distribution of points necessary to constuct a mesh object from Delaunay Triangulation.[3] The surface area of each feature is

then calculated as the sum of the areas of each triangle within the triangular mesh generated.

Aspect ratios were also calculated by utilizing the trimesh figures generated of each blister. Each aspect ratio was calculated using h/r where h is the maximum blister height and r is the blister radius from that point. This was accomplished by finding the maximum height of a masked nanoblister, then centering the blister trimesh at point h around a graphical origin (0, 0 , 0). The maximum value of X was then taken as the blister radius. All radii were measured in the X direction to consistently sample blister dimensions. As such, oblong-shaped blisters were aligned accordingly to measure the shorter radius along the X axis. The aspect ratio analysis becomes less accurate with less circular shaped blisters.

Micro-FTIR Spectroscopy

Infrared spectral data was recorded as absorbance values, log(1/R), and exported from the instrument as comma-separated values. The collected spectra were the average of 128 scans measured with 1 cm^{-1} resolution, and all data were processed using Python 3.9 scripts written using the Spyder v.5.3.3 IDE. Spectra were processed with a 25-point second-order (parabolic) Savitzky-Golay filter to remove perturbations from gaseous H_2O signals in the air between the objective and the sample. While the Savitzky-Golay filter improves the analysis of intensity information at the cost of frequency information, the 1 cm^{-1} resolution allowed for confident extraction of frequency values from spectra.

AFM Image Processing and Characterization

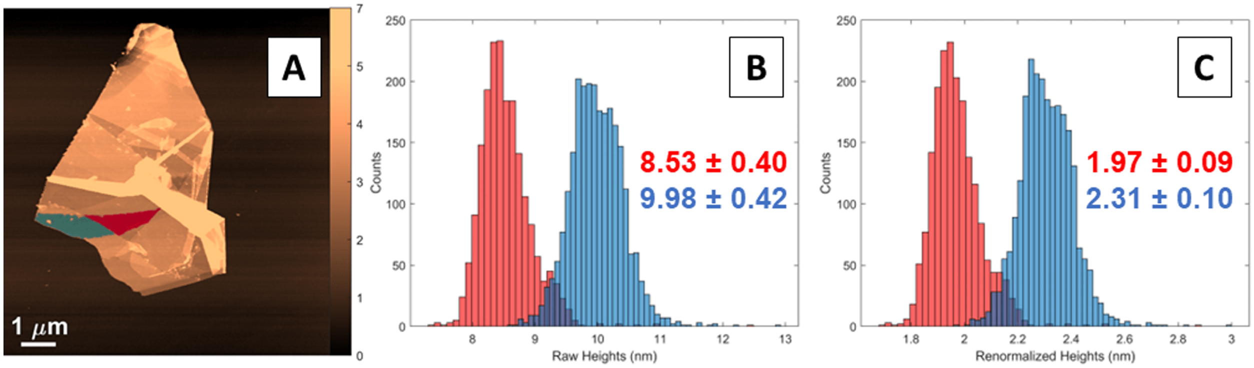

Figure S2: A large few layer graphene flake demonstrating internal calibration by an FLG step and the phase contrast observed between mica and graphene. (A) AFM topography image 10 μm $\times$ 10 μm after rennormalization, with the upper and lower terraces of an FLG step masked in blue and red, respectively. (B) Raw height distribution for the masked upper and lower terraces. (C) Height distribution for the masked upper and lower terraces after height renormalization. The mean $\pm$ one standard deviation is reported for each terrace by color, and the standard deviation represents the pixel to pixel height variation within the respective masked region.

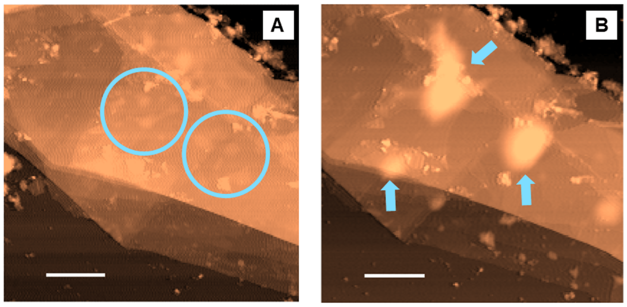

Figure S3: AFM topographs (5 μm × 5 μm) depicting consolidation of water features into nanoblisters. (A) The as deposited flake with morphological features indicative of confined water, such as the features within the regions circled in blue. (B) The same flake after a 120 °C anneal, where the consolidation of water into large nanoblisters removed the confined water features observed in (A). The blue arrows indicate examples of nanoblisters filled with consolidated water. All scale bars are 1 μm.

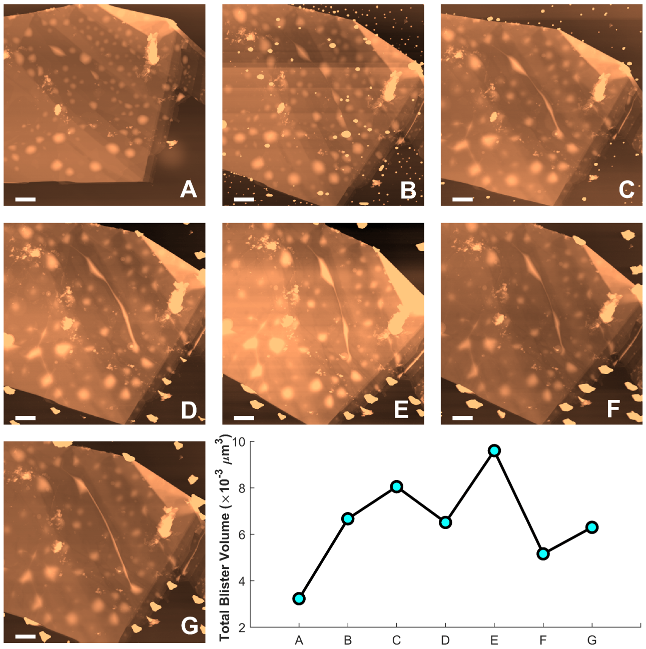

Figure S4: AFM topographs (10 μm $\times$ 10 μm) of the dry/wet annealing cycle, expanding beyond A−C which were included in Fig. 1 of the main text. Panel A depicts the as deposited few-layer graphene flake, panels (B, D, & F) depict dry anneals, and panels (C, E, & G) depict wet anneals. All anneals were performed consecutively in alphabetical order, at 120 °C for 1 hour each. Each scale bar is 1 μm. The linegraph from Fig. 1 is included for reference.

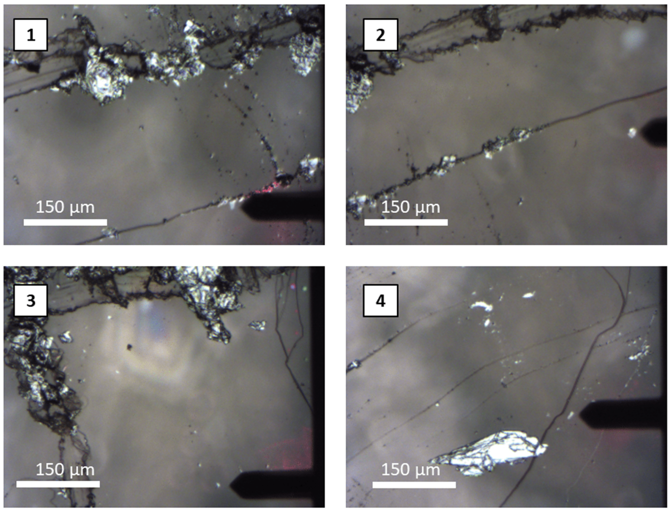

Figure S5: Optical micrographs of few-layer graphene flakes used for FTIR spectra. Spectra from flakes 1 and 2 were used in Fig. 3 of the main text. The AFM probe tip points to each flake analyzed.

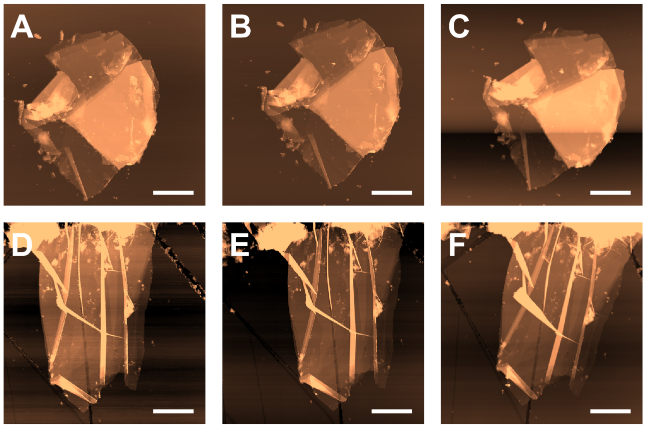

Figure S6: 20 μm $\times$ 20 μm AFM height images of the flakes used for FTIR spectra in Fig. 3. Each scale bar is 4 μm. (A&D) are as deposited, (B&E) are after dry annealing for 1 hour at 120 °C, (C&F) are after wet annealing for 1 hour at 120 °C.

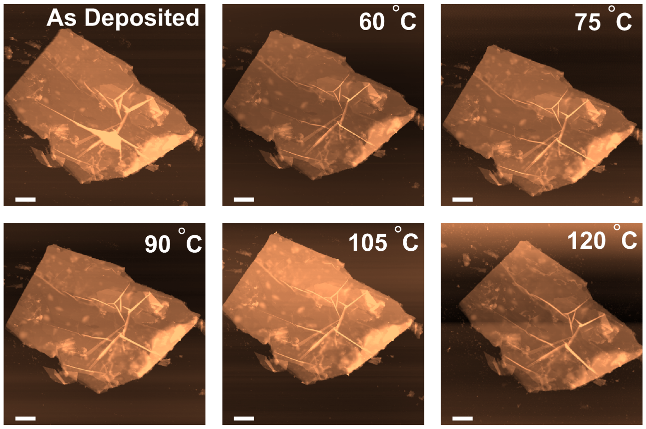

Figure S7: The full AFM height images (13 μm × 13 μm) corresponding to the temperature annealing series in Fig. 4. Each scale bar is 2 μm, and annealing temperatures are indicated.

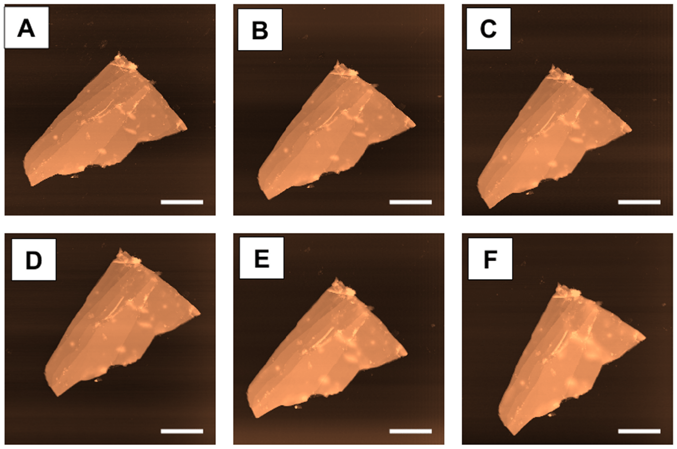

Figure S8: The full AFM height images (10 μm × 10 μm) corresponding to the isothermal annealing series in Fig. 5. Each scale bar is 2 μm, and all anneals were performed at 60 °C. Panel A depicts the as deposited flake, and panels B−F depict the flake after 1, 2, 4, 8, and 16 total hours of annealing, respectively.

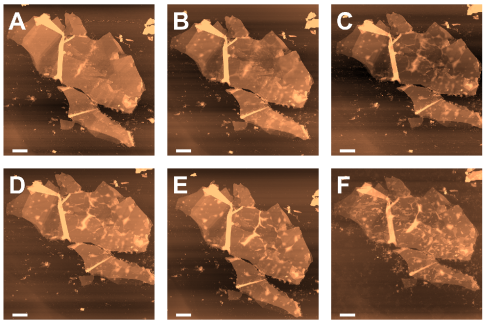

Figure S9: The 20 μm $\times$ 20 μm AFM images of the few layer graphene flakes analyzed for use in Table 1 of the main text. Each scale bar is 2 μm. Panel A depicts the flake as deposited, and panels B−F depict the flake after wet annealing at 60, 75, 90, 105, and 120 °C, respectively.

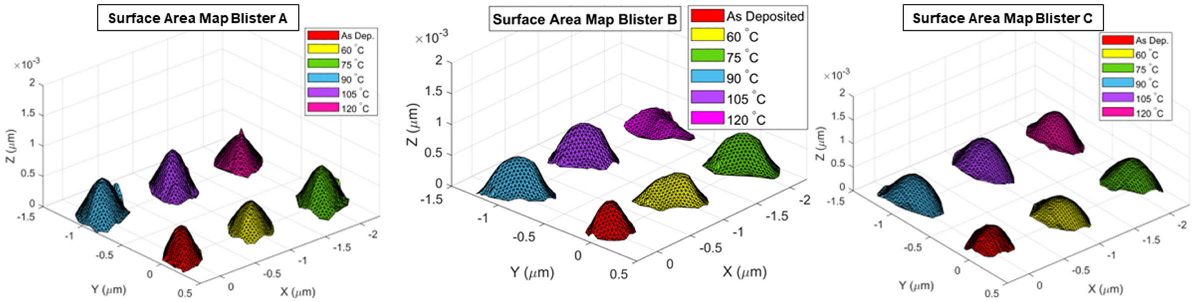

Figure S10: Surface area analysis of blisters A, B, and C indicated in the Varied Temperature Series from Fig. 6 of the main text. The legends included detail the annealing condition of each trimesh blister shown

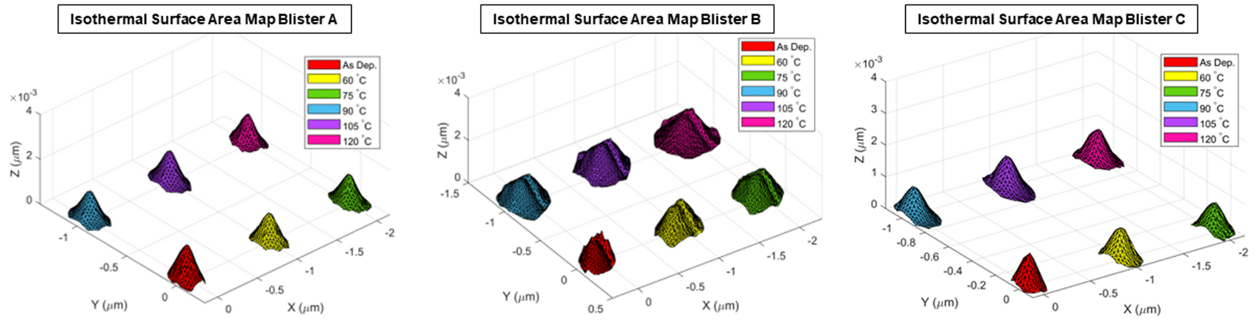

Figure S11: Surface area analysis of blisters A, B, and C indicated in the Isothermal Series from Fig. 6 of the main text. The legends included detail the annealing condition of each trimesh blister shown.

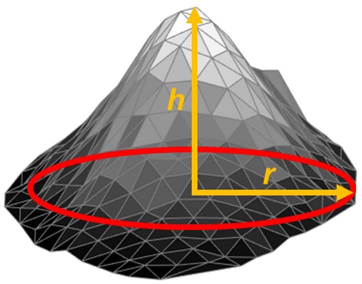

Figure S12: Determination of the aspect ratio for a nanoblister. The blister height h was measured from the blister maximum, and the radius r was measured from the h to the nearest side. Both h and r are depicted in orange, and the circle formed by r is shown in red.

References

1. Bridgeman, O. C.; Aldrich, E. W. Vapor Pressure Tables for Water. *J. Heat Transf.* **1964**, *86*, 279–286.

2. Xu, K.; Cao, P.; Heath, J. R. Graphene Visualizes the First Water Adlayers on Mica at Ambient Conditions. *Science* **2010**, *329*, 1188–1191.

3. Delaunay, B. “Sur la sphère vide, Izvestia Akademii Nauk SSSR, Otdelenie Matematicheskikh i Estestven- nykh Nauk,”. *Sci. Res. Publ.* **1934**, 793–800.
